# Interaction between Trehalose and MTHase from *Sulfolobus solfataricus* Studied by Theoretical Computation and Site-Directed Mutagenesis

**DOI:** 10.1371/journal.pone.0068565

**Published:** 2013-07-19

**Authors:** Chien-wei Fu, Yu-Ping Wang, Tsuei-Yun Fang, Thy-Hou Lin

**Affiliations:** 1 Institute of Molecular Medicine and Department of Life Science, National Tsing Hua University, Hsinchu, Taiwan; 2 Department of Food Science and Center of Excellence for Marine Bioenvironment and Biotechnology, National Taiwan Ocean University, Keelung, Taiwan; Wake Forest University, United States of America

## Abstract

Maltooligosyltrehalose trehalohydrolase (MTHase) catalyzes the release of trehalose by cleaving the α-1,4-glucosidic linkage next to the α-1,1-linked terminal disaccharide of maltooligosyltrehalose. Computer simulation using the hydrogen bond analysis, free energy decomposition, and computational alanine scanning were employed to investigate the interaction between maltooligosyltrehalose and the enzyme. The same residues that were chosen for theoretical investigation were also studied by site-directed mutagenesis and enzyme kinetic analysis. The importance of residues determined either experimentally or computed theoretically were in good accord with each other. It was found that residues Y155, D156, and W218 of subsites -2 and -3 of the enzyme might play an important role in interacting with the ligand. The theoretically constructed structure of the enzyme-ligand complex was further validated through an *ab initio* quantum chemical calculation using the Gaussian09 package. The activation energy computed from this latter study was very similar to those reported in literatures for the same type of hydrolysis reactions.

## Introduction

**Figure 1:**
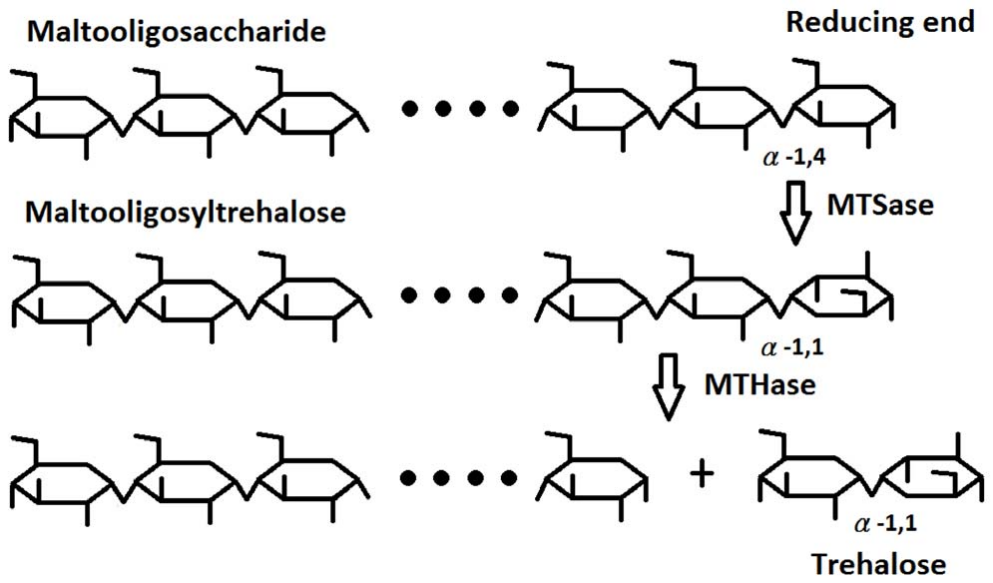
Production of trehalose from maltooligosaccharide via MTSase and MTHase. In the first step, MTSase turns the α-1,4-linked terminal disaccharide of maltooligosaccharide into the α-1,1-linked terminal disaccharide. In the second step, MTHase cleaves the α-1,4-glucosidic linkage next to the α-1,1-linked terminal disaccharide of maltooligosyltrehalose and produces a trehalose.

Trehalose (α-D-glucopyranosyl-α-D-glucopyranoside) is a non-reducing sugar formed from two glucose (G_1_) units joined by an α-1,1 linkage. Because trehalose can protect proteins and lipid membranes from desiccation, freezing, high temperature, and osmotic stress, the sugar has been applied to many different areas, such as the use as a preservative or stabilizer for cells, organs, food, cosmetics, and medicines [1]. The sugar has also been approved as a novel food ingredient under the GRAS term in the United States and Europe [2]. Trehalose is released mainly from cleavage of the α-1,4-glucosidic linkage next to the α-1,1-linked terminal disaccharide of maltooligosyltrehalose by maltooligosyltrehalose trehalohydrolase (EC 3.2.1.141, MTHase). Trehalose can also be produced from starch by a combined enzymatic treatment using the thermophilic maltooligosyltrehalose synthase (MTSase), MTHase, and a debranching enzyme (Figure 1). However, both MTSase and MTHase catalyze a side hydrolysis reaction which would decrease the yield of trehalose [3]–[5]. The structure of MTHase from *Sulfolobus* (*S.*) *solfataricus* KM1 is organized by three major domains namely, A, C, and E and two subdomains B and D. Domain A of MTHase of *S. solfataricus* KM1 is the catalytic domain made by a (*β*/α)_8_ barrel where both subdomains B and D are protruded from this barrel [6].

It has been shown previously that the catalytic mechanism of MTHase from *S. solfataricus* KM1 involves in the following three carboxyl groups namely, D252, E283, and D377 [5], [6]. By comparing the structures of MTHase from *S. solfataricus* KM1 and several other glycosidases from family 13 of glycosyl hydrolases, it is found that the trehalose end of maltooligosyltrehaloses is most likely to bind with the +1 and +2 subsites of the enzymes which are located near the C-terminus of the catalytic barrel. While binding with subsite +1 is to provide a recognition for the trehalose moiety, the most critical region for substrate discrimination between maltooligosyltrehaloses and maltooligosaccharides is subsite +2 [6]. It is known that formation of hydrogen bond is important to the mechanism of enzymatic catalysis. The substrate specificity and the rate of enzymatic reactions are directly influenced by the formation of hydrogen bonds between the enzyme and its substrate. In other MTHases, a previous structure study has demonstrated that a hydrogen bond network and some hydrophobic interactions are formed between trehalose and subsites +1 and +2 of MTHase of *Deinococcus radiodurans* (DrMTHase) [7]. In DrMTHase, H332 can recognize the α-1,1-glycosidic linkage of the trehalose molecule by forming hydrogen bonds with the O6 atoms on both sugar rings. While both H332 and E376 are forming hydrogen bonds with the ring at subsite +2, N404 is forming hydrogen bonds with the ring in subsite +1 [7]. The ring in subsite +2 is also being stabilized by D328, D329, R380, and Y349 via contacts with the water molecules [7]. This study [7] then reveals that trehalose only occupying subsites +1 and +2 of the DrMTHase structure. There may be some other interactions between the substrate and other subsites of MTHase which could be assessed only when the structures of both MTHase-maltooligosyltrehalose and MTHase-maltooligosaccharide complexes will be solved.

**Figure 2:**
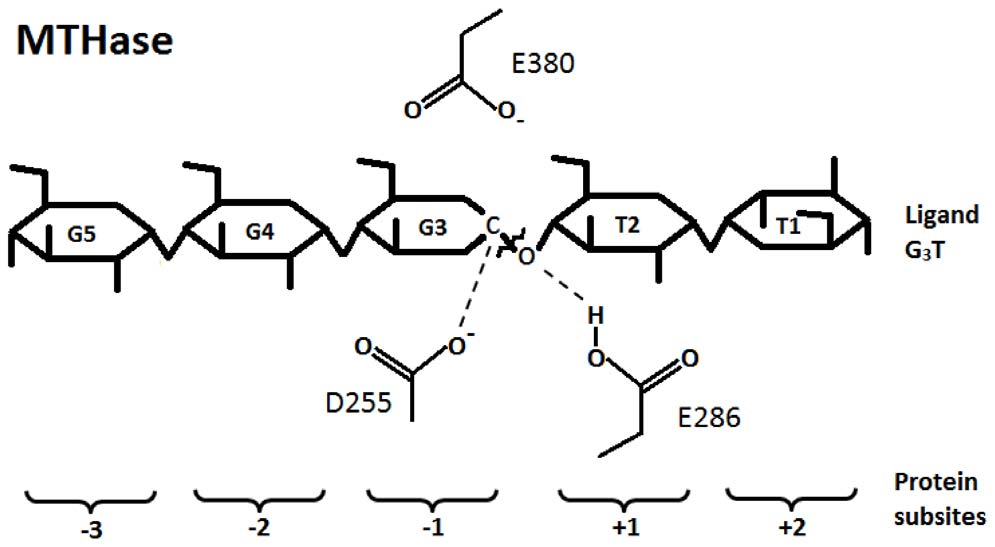
Production of trehalose from ligand G_3_T by MTHase. Ligand G_3_T is a maltooligosyltrehalose consisting of five glucoses. The first four glucoses are α-1,4-linked while the terminal two ones are α-1,1-linked. The glucoses of G_3_T are numbered from G5 to T1. The catalytic triad residues identified in MTHase are D255, E286 and E380, respectively. The protein subsites are numbered from +3 to −2. The bond between T2 and G3 is cleaved during the hydrolysis reaction.

The MTHase from *S. solfataricus* ATCC 35092, also known as P2, has been purified and characterized [8]. The deduced amino acid sequence of MTHase from *S. solfataricus* ATCC 35092 possesses a sequence identity of 79.9% to the enzyme from *S. solfataricus* KM1 [8]. In addition, the pocket of active site of MTHase from *S. solfataricus* KM1 [6] are formed by several conserved residues which are similar to that found in MTHase from *S. solfataricus* ATCC 35092 [8], suggesting that these two proteins may interact with the same ligand. Recently, we had conducted a series of mutational analyses on the active site of MTHase of *S. solfataricus* ATCC 35092 and found that residues D255, E286, and D380 (equal to D252, E283 and D377 in MTHase of *S. solfataricus* KM1) may be the essential catalytic residues of MTHase (Figure 2), while residues W218, A259, Y328, F355, and R356 (equal to W215, A256, Y325, F352, and R353 in MTHase of *S. solfataricus* KM1) may be the selectivity-related residues of the enzyme. We had also found that the selectivity ratios of MTHase could be increased by the following mutations A259S, Y328F, F355Y, and R356K while decreased by the mutations W218F and W218A [5]. The selectivity ratios were defined as the ratios of the catalytic efficiencies for formation of glucose to those for formation of trehalose during the hydrolysis of maltooligosaccharides and maltooligosyltrehaloses by the enzyme, respectively.

The structural and energetic details of interactions between biomolecules especially the contribution from different energetic terms such as electrostatic, van der Waals, as well as solvation energy to the binding can be evaluated through the theoretical computation [9]. The solvation energy is usually computed either from the Molecular Mechanics generated Poisson Boltzmann Surface Area (MM-PBSA) or Generalized Born Surface Area (MM-GBSA) [10]–[15]. While the electrostatic contribution to the solvation term of the former is computed through solving the Poisson-Boltzmann equation, that of the latter is computed from solving the Generalized Born (GB) equation. Both methods have been implemented in some molecular dynamics (MD) simulation packages for studying the interaction between biomolecular complexes using the explicit solvent models [9], [16]–[24]. The information on contribution from each individual residue or atom to the overall binding free energy of a wild type protein complex can also be obtained through free energy decomposition. Using the computational alanine scanning, the difference in binding free energy between a wild type protein complex and an Ala-mutated protein one can also be computed. For examples, both the solvation contribution to binding modes at the atomic level and the protein stabilities have been evaluated using the free energy decomposition with either the MM-GBSA [16], [25]–[27] or MM-PBSA method [16], [24], [27]–[33]. Here, the same theoretical computation methods are employed to investigate the mechanism of hydrolysis of maltooligosyltrehalose (designated as G_3_T hereafter) by MTHase. The trehalose is released from cleavage by MTHase at the α-1,4-glucosidic linkage next to the α-1,1-linked terminal disaccharide of maltooligosyltrehalose. The site-directed mutagenesis was also performed on the same residues studied by the theoretical methods. The difference in binding free energy between each of the wild-type and mutated residue determined experimentally was compared with those computed theoretically. An *ab initio* quantum chemical calculation using the static structural model built was also conducted to validate the theoretically constructed structure of the enzyme-ligand complex.

## Methods

### Materials of Mutagenesis

*E. coli* BL21-CodonPlus (DE3)-RIL was purchased from Stratagene (La Jolla, CA). Maltopentaose (G_5_), 3,5-dinitro salicyclic acid (DNS), ampicillin, chloramphenicol, streptomycin sulfate, and bovine serum albumin (BSA) were supplied by Sigma (St. Louis, MO). Q-Sepharose and protein low-molecular-weight standards were purchased from Amersham Pharmacia Biotech (Piscataway, NJ). Maltotriosyltrehalose (G_3_T) was prepared from G_5_ as previously described [8].

### Site-directed mutagenesis

The MTHase gene in the previously constructed vector pET-15b-ΔH-*treZ* [8] was mutated by polymerase chain reaction (PCR) according to a megaprimed and ligase-free PCR-based site-directed mutagenesis method [34]. The designed mutations were included in the mutagenic primers which have 27 to 41 bases individually.

### Production and purification of MTHase

Wild type and mutant (Y155A, Y155F, D156A, H195A, R447A, and E450A) MTHases were produced by culturing the vector pET-15b-ΔH-*treZ* transformed *E. coli* BL21-CodonPlus (DE3)-RIL strain at 37°C in a terrific broth supplemented with 100 ug/mL ampicillin plus 34 ug/mL chloramphenicol as previously described [8]. The cell free extracts containing wild type and mutant MTHases were prepared by using a French Press, centrifugation, and then heat treatment in a 80°C water bath for 1 h to remove the heat-labile proteins. The unwanted nucleic acids were removed through precipitation using 1% (w/v) streptomycin sulfate added as described previously [8]. Both the wild type and mutant MTHases were then purified using a Q-Sepharose anion exchange column (1.6×10 cm) pre-equilibrated in a 20 mM Tris-HCl buffer (pH 8.5). After loading the sample, the column was completely washed with the same buffer until the absorbance at 260 nm dropping to the baseline. Finally, a linear gradient of 0 – 2 M NaCl in the aforementioned buffer was used to elute the bound proteins. The eluted fractions with detectable enzymatic activity were pooled together and then dialyzed against the same buffer. The protein concentration was determined by the Bradford method [35] using BSA as the standards.

### Enzyme kinetics

The initial rates for hydrolysis of G_3_T were determined from using 8 to 10 substrates with concentrations ranging from 2 to 50 mM in a 50 mM citrate-phosphate buffer at pH 5 and 60°C as described previously [8]. The values of *k*_cat_, *K*_M_ and *k*_cat_/*K*_M_ were calculated by fitting the initial rates as a function of substrate concentration to the Michaelis-Menten equation using the Enzfitter software (Elsevier-Biosoft). The standard errors of these parameters were obtained from the fitting results. The change in transition state binding free energy ΔΔG for hydrolysis of the ligand by the mutant enzymes was used to estimate the binding strength of the ligand in the transition state complex and was calculated through the equation ΔΔG  =  −*RT*ln[(*k*_cat_/*K*_M_)_mut_/(*k*_cat_/*K*_M_)_wt_], where the subscripts mut and wt were used to denote the mutant and wild type enzyme, respectively [36].

### Set up the system for theoretical computation

The structure of MTHase of *S. solfataricus* KM1 determined with 3.00 Å resolution by X-ray crystallography (PDB code 1EH9) is a dimer composed by two chains A and B [6]. Each of these two chains containing 558 residues and is linked by a disulfide bridge between the two Cys298 residues of 1EH9. Both of these two chains were included in the MD simulation to keep the protein structure stable. The AMBER 11 suite of programs [37]–[39] were used to conduct all the MD simulations. Because 1EH9 is an apo enzyme where a ligand in its active site is lacking, we utilized the molecular docking program GOLD [40], [41] to dock the theoretically generated G_3_T structure into its active site to obtain the initial ligand conformation. The information of active site residues was obtained from previous studies of MTHase [5], [6] and catalytic mechanisms of some other similar glycosylases [42]. However, the protonation states of other residues were defined using the two web available methods H++ [43] and PROPKA [44]. Previous crystal structure and mutagenesis studies had demonstrated that D255, E286, and D380 of MTHase of *S. solfataricus* ATCC 35092 were the essential catalytic residues of the catalytic triad, and Y328, F355, and R356 were the residues of subsite +2 of the active site [5], [6]. These information helped us to dock G_3_T into the active site in an appropriate way.

The force fields ff99SB [45] and GLYCAM06g [46] were employed during the MD simulations as the parameters for protein and ligand G_3_T, respectively. The protein-ligand complex was hydrated in a box of explicit TIP3P water molecules [47], [48] of a dimension of 10Å from the protein margin. The total water molecules used were 36285 and there were also 38 Na^+^ and 12 Cl^−^ ions included to neutralize the system. The system was first minimized by 1000 steps of conjugate gradient to remove bad atomic contacts within the protein and ligand structures. A non-bonded cutoff distance of 12 Å was applied throughout the MD simulation runs. The system was then gradually heated from 0 to 300 K in 500 ps by restraining all the Cα atoms of the protein with a restraining force of 10 kcal/mol/Å^2^. A subsequent 500 ps simulation run was performed at a constant temperature of 300 K to achieve a constant pressure of 1 atm. Thereafter, the restraints of Cα atoms of the protein were removed and the system was equilibrated for an additional 3 ns period. After the equilibration phase of the system had been completed, the system was run further for 20 ns of the production period using the PMEMD module of AMBER 11 and the trajectory of the last 10 ns of MD simulation was analyzed respectively using the PTRAJ module of AMBER 11 and AMBER Tools 1.5 [39]. The time step of MD simulation was set as 2 fs and the periodic boundary condition was applied to all the dimensions of the simulation box. The Particle Mesh Ewald (PME) method [49] was used to compute the energy of long-range electrostatic interactions and the SHAKE method [50] was used to constrain all the covalent bonds involving the hydrogen atoms.

### GBSA and PBSA

The MM-PBSA and MM-GBSA methods [10]–[15] were employed to compute the solvation energy term of the binding free energy of the protein-ligand complex. There were about 1000 snapshots taken with an interval of 10 ps from the last 10 ns of the MD trajectory for analyzing the binding free energy. The binding free energy is given as follows:
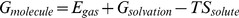
(1)where G_solvation_ is the solvation free energy of the molecule and E_gas_ is the gas phase molecular mechanical energy of the solute obtained by summing the internal ( E_int_), electrostatic (E_ele_), and van der Waals ( E_vdw_) interaction energies. TS_solute_ is the entropy term summed from TS_trans_, TS_rot_, and TS_vib_ which are the translational, rotational, and vibrational entropy contributions, respectively. The entropy term of TS_vib_ is determined using the NMODE module of AMBER 11 via the normal mode analysis involving the calculation and diagonalization of a mass-weighted second derivative matrix. The solvation free energy G_solvation_ is calculated by a GB/SA model where the energy is divided into an electrostatic (G_gb_) and a non-polar (G_np_) component. The MM-GBSA method is briefly summarized as follows:
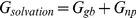
(2)where G_gb_ is the electrostatic contribution to the solvation free energy and is computed from the continuum generalized Born solvent model and G_np_ is the non-polar solvation term computed from the solvent accessible surface area (SASA) using the Linear Combination of Pairwise Overlaps (LCPO) method implemented in the AMBER 11 package [16], [25]–[27]. For the MM-PBSA method, the polar solvation term G_gb_ is replaced by G_pb_ [16], [24], [27]–[32]. The G_solvation_ is calculated with a PB/SA model which is summed from the electrostatic (G_pb_) and non-polar component (G_np_). The binding free energy G_bind_ of the MTHase is then given as:
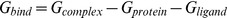
(3)Where G_complex_, G_protein_, and G_ligand_ stand for the free energies computed for the complex, protein, and ligand, respectively. The binding free energy can be computed using either the separate trajectory (three different trajectories of the complex, protein, and ligand, respectively) or from single trajectory protocol (a trajectory of the complex). In this work, we used the single trajectory protocol to evaluate the free energy differences of many mutations [23], [24]. As has been demonstrated by Massova and Kollman [28], the entropic contribution to the relative difference in binding free energy computed between the wild type and a mutant protein with only one mutated residue could be minor. The major thermodynamic factor could be driven from the enthalpy of interaction between the protein and ligand [28]. Therefore, the entropic contribution to the relative difference in binding free energy computed was not considered here.

### Alanine scanning

To evaluate the importance of active site residues, we applied the alanine scanning method to estimate the relative binding affinity of different mutants to ligand G_3_T. The alanine mutant structures were generated by altering the side chains of the wild-type residues. This mutation method involves removing all side chain atoms except atom C_β_ [28] and the parameters for mutated residues were replaced by the alanine residue. We used the same 1000 snapshots taken from the last 10 ns of the MD trajectory with a time interval of 10 ps for performing the alanine scanning. The residues in subsites −1 to −3 of the active site of MTHase namely, Y155, D156, H195, W218, R447, and E450 were chosen for performing the alanine scanning.

### *Ab Initio* Quantum Chemical Calculation with the Truncated Model System

Both the binding structure of G_3_T with MTHase and the mechanism of hydrolysis on ligand by the enzyme were investigated using the *ab initio* quantum chemical calculation through the Gaussian09 package. The truncated model used for quantum chemical calculation was constructed using the structures generated from the MD simulations by the AMBER package. The key MTHase residues in subsites +1 to −1 and the ligand were retained and extracted from the final structures of MD simulation. To construct the truncated model system, the structures of key residues were curtailed at the alpha carbon atom. Both the carbon and nitrogen atoms at the truncating boundary were replaced by hydrogen ones. The total number of atoms of the truncated system was 142. The positions of terminated carbon atoms were also fixed during the geometry optimization by assuming that these atoms were moving insignificantly during the reaction. The geometry optimization was performed using the restricted Hartree-Fock (HF) method with the 3–21G basis set. The 6−31G basis set was used for the refinement of energy and geometry estimated on each stationary point. The self-consistent reaction field calculation was performed to account the influence of surrounding on the protein and environment inside the protein. The PCM model was employed to evaluate the solvation energy of the active site of the protein.

## Results

### Purification and thermostability of wild-type and mutant MTHases

**Figure 3:**
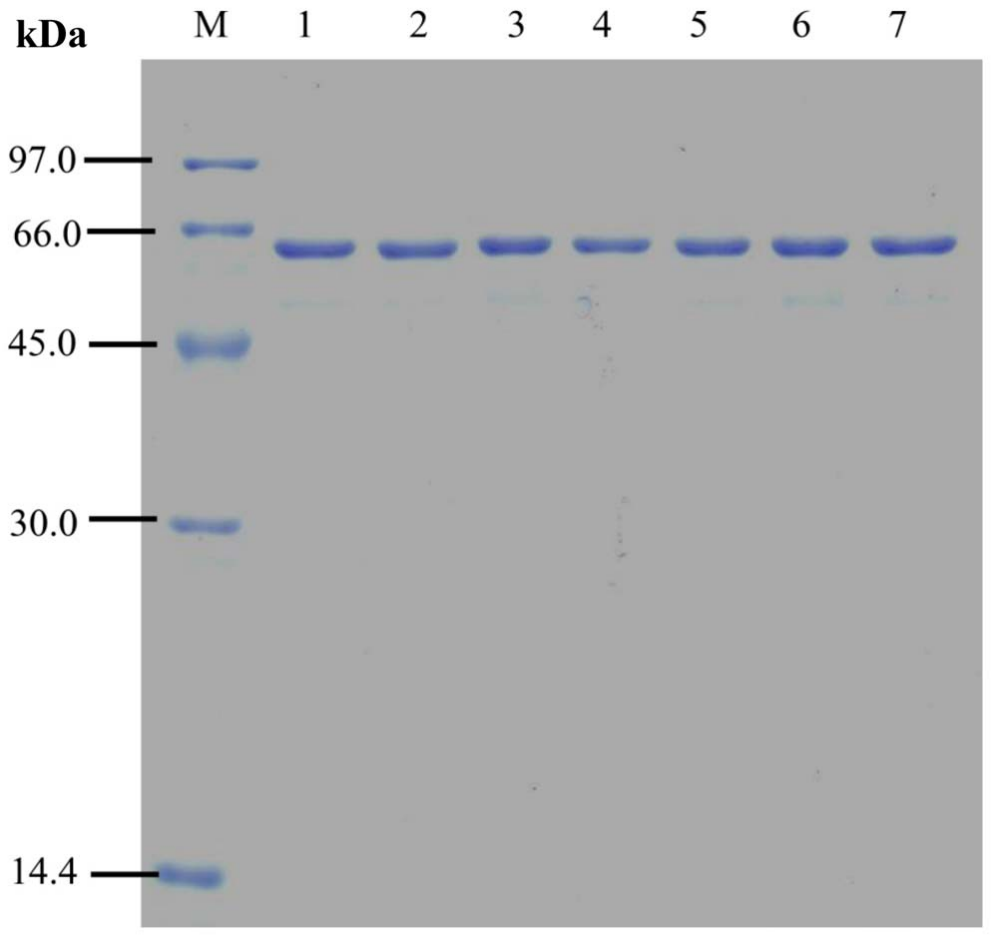
SDS-polyacrylamide gel electrophoresis of the purified wild type and mutant MTHases. The purified wild type and mutant MTHases were analyzed by a 12% minigel and stained with Coomassic Brilliant Blue R-250. Lane M: the molecular weight standards; lanes 1−7: 2.2 µg of wild-type, Y155A, Y155F, D156A, H195A, R447A, and E450A MTHases, respectively.

The purities of wild type and mutant MTHases prepared were good as shown by the SDS-PAGE analysis (Figure 3). In fact, the purities of all the purified wild type and mutant MTHases estimated using GelAnalyzer 2010 [51] were greater than 97%. The residual activities of purified wild type and mutant MTHases measured were 87.4 ± 0.3 (wild type), 91.8±2.4 (Y155A), 111.7±0.4 (Y155F), 110.4±0.8 (D156A), 84.6±0.9 (H195A), 100.1±2.2 (R447A), and 104.9±1.4 (E450A) %, respectively, when they were all incubated at 80°C for two hours. This implied that the mutant enzymes were as thermostable as the wild type one.

### Enzyme kinetics

**Figure 4:**
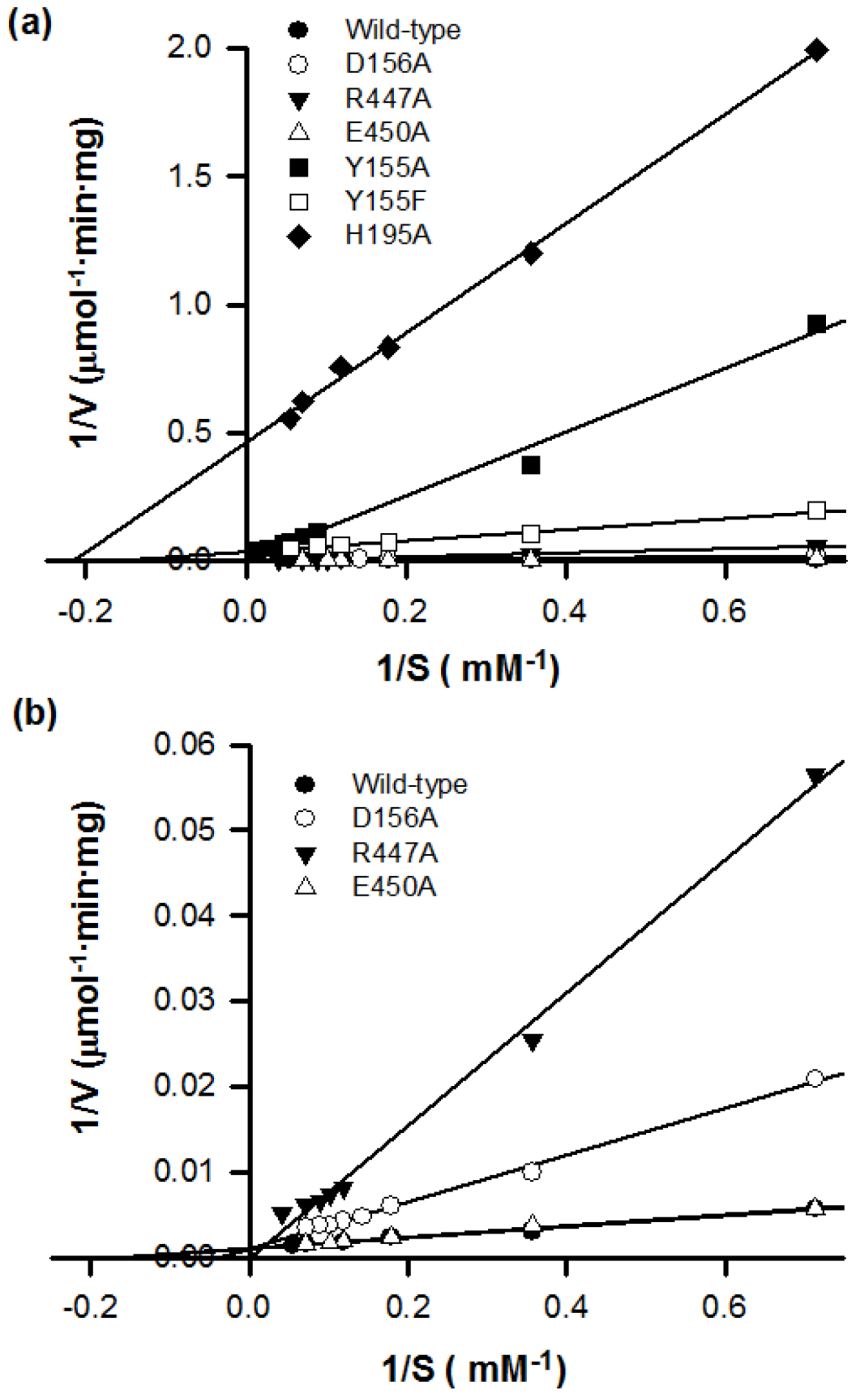
The Lineweaver-Burk plots generated for both wild type and mutant MTHases. In panel (a), the Lineweaver-Burk plots for the wild type and Y155A, Y155F, and H195A mutant MTHases were plotted. In panel (b), the Lineweaver-Burk plots for the wild type, D156A, R447A, and E450A mutant MTHases were plotted.

**Figure 5:**
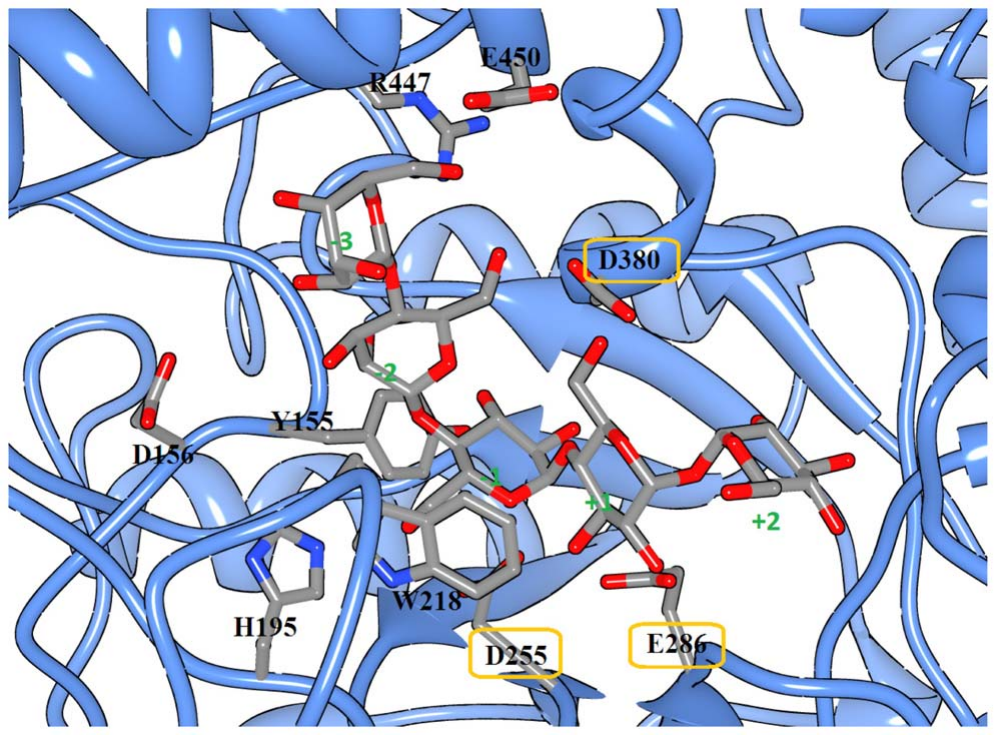
The active site residues of MTHase and ligand G_3_T. Ligand G_3_T is shown in stick while MTHase is shown in light blue ribbon. The key MTHase residues are also shown in stick. Oxygen atoms are colored in red, nitrogen atoms are colored in blue, and carbon atoms are colored in grey. The residues of catalytic triad are highlighted with yellow circles. The subsites of NTHase are numbered from +2 to −3.

The kinetic parameters (*k*_cat_ and *K*_M_) for the hydrolysis of G_3_T at 60°C and pH 5 were shown in Table 1. The activities of Y155A, Y155F and H195A MTHases were low as compared with those of the wild type one (Table 1 and Figure 4). The catalytic efficiency can be conveniently determined as the reciprocal of the slope of a Lineweaver-Burk plot shown in Figure 4. As shown in Table 1, much lower *k*_cat_ and higher *K*_M_ values were determined for the Y155A MTHase, while much lower *k*_cat_ and similar *K*_M_ values than that of wild type one were determined for the Y155F MTHase. As a result, the catalytic efficiencies of Y155A, Y155F, and H195A MTHases estimated were 0.63, 3.04, and 0.28% of that of wild type MTHase, respectively (Table 1 and Figure 4). Though not being as severely impaired as those of the Y155A, Y155F, and H195A mutant enzymes, the catalytic efficiencies determined for R447A, D156A, and E450A mutant enzymes were respectively 15, 26, and 79% of that of the wild type one (Table 1 and Figure 4). The difference in binding strength of an enzyme-substrate complex in the transition state between the wild type and mutant MTHases was also estimated to examine whether a mutation would change the binding between the enzyme and substrate or not. Some earlier studies have shown that the change in transition state energy ΔΔG associated with the loss of a hydrogen bond between the uncharged groups of substrate and enzyme is about 0.5 to 1.5 kcal/mol, while that for loss of a hydrogen bond between an uncharged group of substrate and a charged group of enzyme is from 3.5 to 4.5 kcal/mol [5], [49]. Here, the ΔΔG values determined for the hydrolysis of G_3_T by H195A, Y155A, and Y155F mutant MTHases were 3.87, 3.35, and 2.31 kcal/mol, respectively (Table 1). These suggested that residues H195 and Y155 of subsites −3 and −2 (Figure 5) of the enzyme may interact significantly with the ligand during the hydrolysis process.

**Table 1:** The kinetic parameters measured for the generation° of trehalose from hydrolysis of G_3_T by both wild-type and mutant MTHases at 60°C in a 50 mM citrate-phosphate buffer at pH 5.

MTHase form	*k*_cat_ (s^−1^)	*K*_M_ (mM)	*k*_cat_/*K*_M _(s^−1^ mM^−1^)	ΔΔG^b^ (kcal/mol)
Wild-type	947±40^a^	5.22±0.59	182±14	
Y155A	44.8±1.7	39.2±2.8	1.14±0.04	3.35
Y155F	27.8±0.7	5.02±0.34	5.54±0.25	2.31
D156A	608±62	12.9±2.3	47.3±3.7	0.890
H195A	2.15±0.05	4.18±0.31	0.510±0.03	3.87
R447A	303±16	10.7±1.3	28.2±2.0	1.23
E450A	1070±50	7.48±0.70	144±8	0.160

a Standard error.

b Change of the transition state energy: ΔΔG =  −*RT*ln[(*k*_cat_/*K*_M_)_mut_/(*k*_cat_/*K*_M_)_wt_] [36].

### Computational analysis with MD simulation

**Figure 6:**
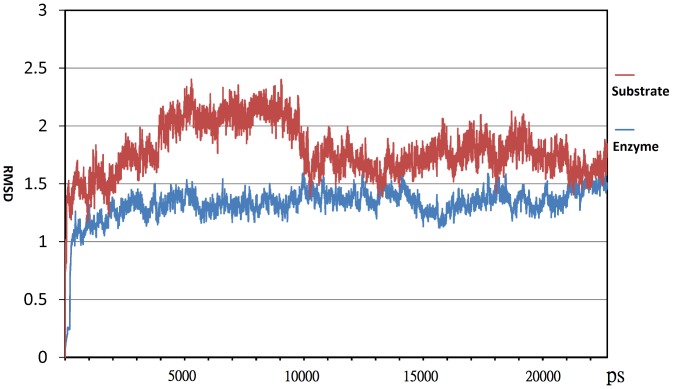
The root-mean-square deviations (RMSDs) of MTHase backbone atoms (blue curve) and all the G_3_T (substrate) atoms (red curve) computed during the MD simulation.

As shown in Figure 2, the distance between atom C1 of ring G3 and a carboxylic oxygen atom of D255 and that between the connecting atom O4 of ring T2 and G3 of G_3_T and a hydrogen atom from the carboxylic group of E286 were both fixed at 3.5 Å during the docking process. The docked conformation of ligand G_3_T was such that rings T1, T2, G3, G4, and G5 of G_3_T were fitted respectively into the subsites +2, +1, −1, −2, and −3 of 1EH9 as shown in Figure 5. Note that most of the important residues studied here namely, Y155, D156, H195, R447, and E450 were located in subsites −2 and −3, respectively (Figure 5). The quality of MD simulation or the stability of MD trajectories generated during 23 ns of MD simulation on the protein-ligand complex was monitored by computing the root-mean-square deviations (RMSDs) of the coordinates of protein backbone atoms from those of the original X-ray determined structure. As shown in Figure 6, a rise in RMSD value at the beginning of simulation was observed and from then on the value was fluctuated around 1.3 Å, indicating that the structure stability during the MD simulation was maintained. As also shown in Figure 6, the RMSD of substrate was also stably maintained during the MD simulation. There were 1000 snapshots taken from the last 10 ns of simulation for performing the free energy computation, alanine scanning analysis, and HB analysis for the wild-type MTHase.

### Binding free energy analyses of wild-type and mutant MTHases

To theoretically explore the role of some suspected active residues of subsites −2 and −3 of MTHase (Figure 5), we performed the HB analysis and computed the binding free energy for the wild-type protein using the PTRAJ module of Amber 11 and Amber Tool 1.5. The role of these residues was also studied using the alanine scanning technique namely, each of them was theoretically mutated to alanine and then the corresponding change in binding free energy with G_3_T was computed. The difference in binding free energy between the wild type and mutant residues was computed as ΔΔG  =  ΔG_wild-type_ −ΔG_mutant_. A positive value of this ΔΔG computed implies that contribution of the residue to the binding with ligand is favorable while a negative one computed means that it is unfavorable. As mentioned in a previous section, the interactions were driven dominantly by enthalpy so that all the entropic contributions were neglected in the computation.

### Y155

**Figure 7:**
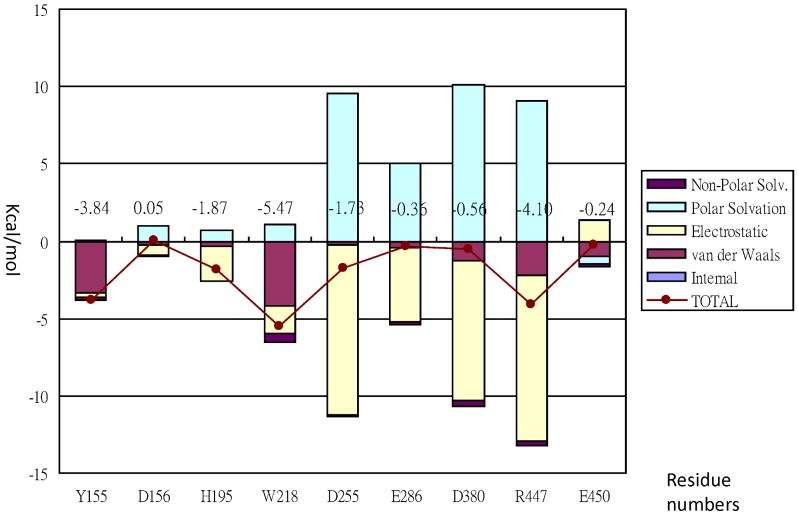
Free energy decomposition of wild type MTHase computed with the MM-GBSA method.

The binding free energy for Y155 with G_3_T computed was −3.84 kcal/mol (Figure 7). This was contributed mainly from the van der Waals (vdw) interaction with G_3_T as a corresponding energy of −3.37 kcal/mol was computed (Figure 7). However, the electrostatic interaction for the residue with G_3_T was rather weak since both a smaller energy of −0.27 kcal/mol (Figure 7) and a smaller occupied time (or %occupied, defined as the snapshots of HB forming/total snapshots [52]) of less than 5% from the HB analysis were obtained for the residue. The difference in binding free energy ΔΔG_subtotal,GB_ and ΔΔG_subtotal,PB_ for mutant Y155A computed from the alanine scanning using both the GB and PB solvation models were 4.37 and 3.48 kcal/mol, respectively (Table 2). The larger the values of ΔΔG_subtotal,GB_ or ΔΔG_subtotal,PB_ computed implying that the stronger the tendency of this residue to interact with the ligand. As shown in Table 1, the experimentally measured ΔΔG from the purified enzyme (Figure 3) and Michaelis-Menten kinetic parameters (Table 1 and Figure 4) was 3.35 kcal/mol. Therefore, both theoretically and experimentally estimated ΔΔG were agreed with each other that Y155 of subsite -2 (Figure 5) might play an important role in interacting with G_3_T.

**Table 2:** The free energy differences computed from the computational alanine scanning for the MTHase mutants.

Substrate G_3_T	W.T.	Y155A	D156A	H195A	R447A	E450A	W218A (ref 5)
Δ**G (kcal/mol) (GB)**	−57.4	−53.0	−57.2	−53.1	−46.2	−57.1	−49.1
Δ**G (kcal/mol) (PB)**	−36.7	−33.2	−39.1	−32.1	−22.2	−36.1	−32.9
Δ**(**Δ**G)(kcal/mol) (GB)**	0	4.37	0.170	4.32	9.75	0.330	8.30
Δ**(**Δ**G)(kcal/mol) (PB)**	0	3.48	−2.39	4.65	15.0	0.630	3.81

### D156

The binding free energy computed for D156 was 0.045 kcal/mol while those computed for both vdw and electrostatic terms were −0.26 and −0.68 kcal/mol, respectively (Figure 7). This may indicate that D156 was not playing a major role in interacting with G_3_T since a occupied time of smaller than 5% was also estimated from the HB analysis for the residue. This was further evidenced by ΔΔG_subtotal,GB_ and ΔΔG_subtotal,PB_ computed from the alaning scanning for mutant D156A where the corresponding values obtained for both parameters were 0.17 and −2.39 kcal/mol, respectively (Table 2).

### H195

Except a occupied time of 75.7% estimated, the binding free energy computed for the residue was −1.87 kcal/mol (Figure 7) which was apparently contributed from the electrostatic interaction with G_3_T. This residue may also play a major role in interacting with G_3_T since the corresponding ΔΔG_subtotal,GB_ and ΔΔG_subtotal,PB_ computed from the alanine scanning for the residue were 4.32 and 4.65 kcal/mol, respectively (Table 2). These also agreed with that obtained experimentally from the mutagenesis studies (Table 1).

### R447

This was the residue where the experimentally determined and theoretically computed results were least agreed with each other. While both binding free energy (−4.09 kcal/mol, Figure 7) and occupied time (75.7%) computed were favoring the interaction with G_3_T, both ΔΔG_subtotal,GB_ and ΔΔG_subtotal,PB_ computed for the residue were huge and were 9.75 and 15.00 kcal/mol, respectively (Table 2). As shown in Figure 7, both the electrostaic and solvation energies computed for this residue were also exceptionally high. These were the causative reasons why the theoretical results computed for the residue were not agreed with those determined experimentally. It was still unclear why the solvation energies computed by both GB and PB models for the residue were extraordinarily high. However, the experimentally determined ΔΔG did show that the strength of interaction of this residue with G_3_T should come after those of H195, Y155, and W218 (Table 2).

### E450

The binding free energy computed for the residue with G_3_T was only −0.24 kcal/mol (Figure 7) which was in parallel with the fact that a smaller occupied time of less than 5% was computed for the residue. Both ΔΔG_subtotal,GB_ and ΔΔG_subtotal,PB_ computed for the residue were 0.33 and 0.63 kcal/mol, respectively (Table 2). These results indicated that the residue was not playing a major role in interacting with G_3_T.

### W218

The binding free energy computed for this residue was −5.47 kcal/mol (Figure 7) which was contributed predominantly from that of the vdw interaction (−4.21 kcal/mol, Figure 7) with the ligand. Except this, the other type of interactions were not so significant as also reflected in a smaller occupied time of less than 5% computed for the residue. However, both ΔΔG_subtotal,GB_ and ΔΔG_subtotal,PB_ computed for the residue were 8.30 and 3.81 kcal/mol, respectively (Table 2), which were in fair agreement with that determined from a previous experimental work (Table 2 and [5]). This would indicate that the residue may also play a major role in interacting with G_3_T.

### *Ab Initio* Quantum Chemical Calculation with the Truncated Model System

**Figure 8:**
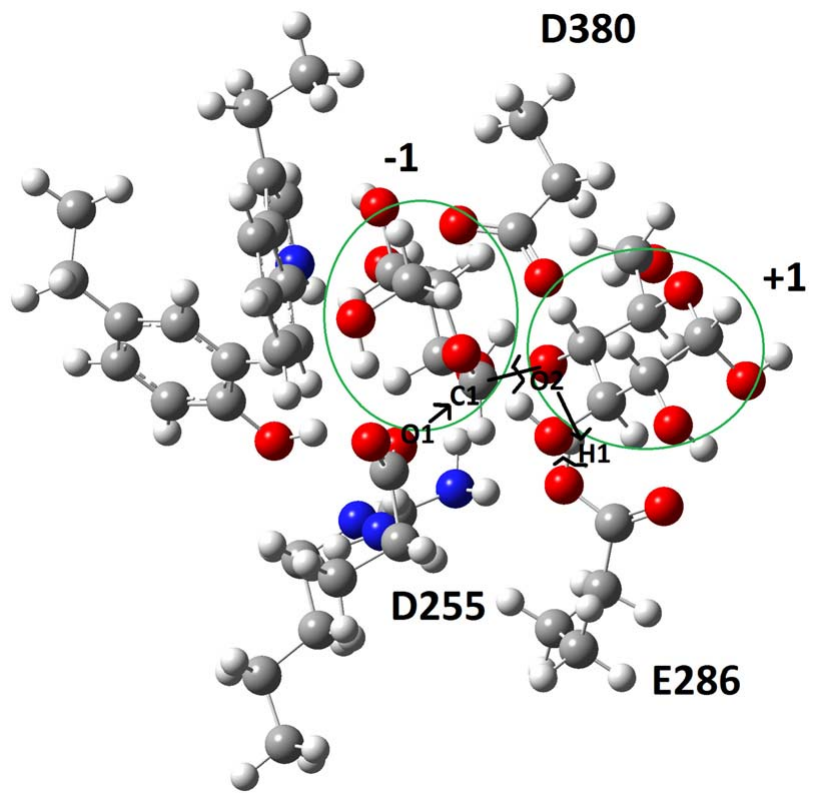
The transition state structure constructed using a truncated model system obtained from the optimized geometries with the 3-21G basis set and Hartree-Fock method. Atoms O1, C1, and O2 represent respectively the oxygen atom of D255 and the carbon and oxygen atom of ligand G_3_T. Atom H1 is representing the hydrogen atom of E286.

**Figure 9:**
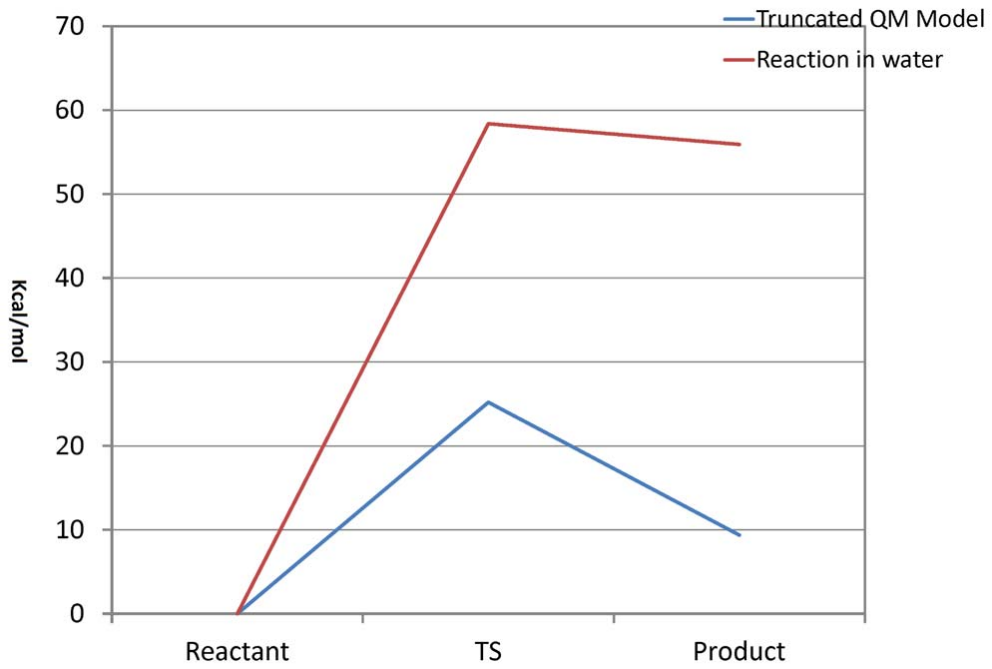
The energy profile of *ab Initio* quantum chemical calculation obtained using the truncated model system for ligand G_3_T in the presence (blue curve) or absence (red curve) of MTHase.

**Figure 10:**
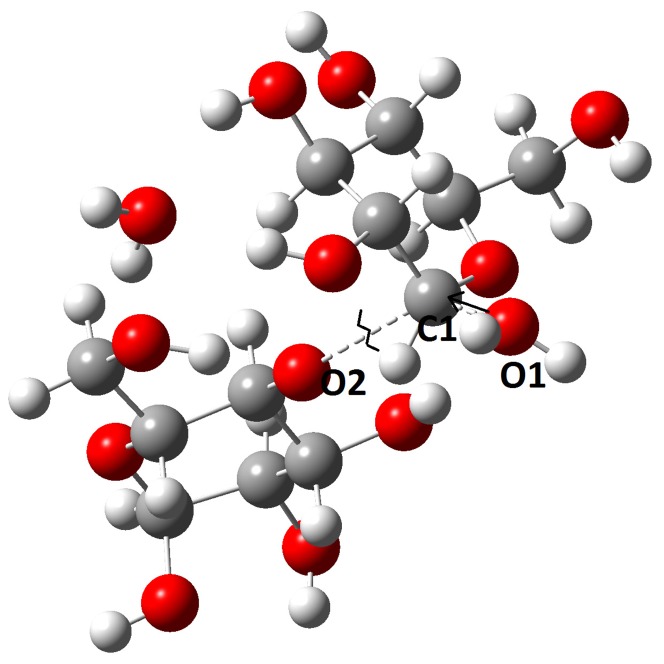
The transition state structure of hydrolysis of G_3_T in the absence of MTHase or in water molecules. Atoms O1, C1, and O2 represent respectively the oxygen atom of a water molecule and the carbon and oxygen atom of ligand G_3_T.

The *ab initio* quantum chemical calculation using the Gaussian09 package is yet an another computational technique that can be used to validate the MTHase-G_3_T model constructed. The computed activation energy and also the hydrolysis products generated would be similar or close to those determined experimentally If the model was correctly constructed. To proceed with Gaussian09 calculation, a truncated model system using optimized geometries with the 3–21G basis set and Hartree-Fock method was built. To refine the geometry and also compute the energy on each stationary point, the HF/6–31G basis set was used. The frequency calculation was used to confirm the energy minima and saddle points searched and only one imaginary vibration mode was allowed for identifying the transition state. The transition state structure identified was further confirmed by the intrinsic reaction coordinate (IRC) calculation. The solvation effect for the presence of water on the system was studied using the PCM model implemented in the package. In general, the hydrolysis reaction catalyzed by MTHase involving D255 acting as a base and E286 acting as an acid. Moreover, as detailed in Figure 8, the reaction was triggered by a proton transfer from the acid (H1 of E286) to the leaving group (O2 of the ligand) and then a nucleophilic attack on atom C1 of the ligand assisted by the base (O1 of D255) was followed. The energy profile of the reaction computed has revealed that there were two minima and one transition state existing in the reaction path (Figure 9). In the first minimum (Reactant), oxygen atom O1 of D255 was moving closer to the carbon atom C1 of the ligand. This first minimum found was characterized by a structure where the O1-C1, C1-O2 and O2-H1 distances determined were 4.86, 1.46 and 1.70 Å, respectively (Figure 8). The highest point of the energy profile (Figure 9) computed was identified as the transition state (TS) of the reaction and was characterized by a tetrahedral structure where the distances determined for O1-C1, C1-O2 and O2-H1 were 2.28, 2.32 and 1.03 Å, respectively (Figure 8). In the second minimum (Product), O1 of D255 was binding with C1 while C1 was departing from O2 of the ligand. The O1-C1, C1-O2 and O2-H1 distances determined for the structure of second minimum (Product) were 1.47, 3.32 and 0.98 Å, respectively (Figure 8). The hydrolysis reaction in the absence of enzyme was also investigated for comparing with that obtained from the truncated QM model. As shown in Figure 10, atom O1 of water molecule was approaching atom C1 of ligand causing the breaking of bond C1-O2 presumably through a nucleophilic attack. The corresponding energy profile of the reaction computed has revealed that there were two minima and one TS existing in the reaction path (Figure 9). The first minimum (Reactant) corresponded to the fact that atom O1 of water molecule was moving toward atom C1 of the ligand. In this minimum, the O1-C1 and C1-O2 distances determined were 3.10 and 1.41 Å, respectively (Figure 10). The highest point in the energy profile (Figure 9) was identified as the TS and the corresponding distances determined for bonds O1-C1 and C1-O2 were 1.74 and 2.58 Å, respectively (Figure 10). However, in the second minimum (Product), atom O1 of water molecule was binding with atom C1 and the corresponding O1-C1 and C1-O2 distances determined were 1.55 and 2.76 Å, respectively (Figure 10).

## Discussion

As revealed by the energy profile (Figure 9) computed, the hydrolysis reaction catalyzed by MTHase on G_3_T was progressed through a typical S_N_2 mechanism. The TS of the reaction was generated as a tetrahedral structure (Figure 8). Upon nucleophilic attack from atom O1 of D255 on atom C1 of the ligand, the C1-O2 bond of ligand was breaking and atom H1 of E286 was then transferred to atom O2 of the ligand (Figure 8). Ligand G_3_T was eventually broken into two parts namely, an intermediate structure formed by D255 with some portion of the ligand through the O1 (D255)-C1 (ligand) linkage (the glycosyl-enzyme intermediate) and a departing glucose moiety (Figure 8). The activation energy of the reaction computed was 25 kcal/mol (Figure 9) which was very close to those (about 20 kcal/mol) have been reported for the same type of hydrolysis reactions [53]–[55]. Thus, this quantum chemical calculation further confirmed that the structure of MTHase-G_3_T complex theoretically constructed was correct. This may be also substantiated by the fact that in the absence of enzyme the same reaction would cost 30 kcal/mol more in activation energy to complete (Figure 9).

In this work, we have employed both experimental and theoretical methods to study the catalytic mechanism for the hydrolysis of trehalose from maltooligosyltrehalose by MTHase. We focused in particular on some residues of subsites −2 and −3 of the enzyme since these are not yet fully explored and the binding structure of the enzyme-ligand complex is still lacking. Residues that were suspected to be important in binding with ligand such as Y155, D156, H195, W218, R447, and E450 were mutated experimentally using the site-directed mutagenesis as well as theoretically using the alanine scanning technique implemented in the AMBER 11 package. Except ΔΔG of R447A computed through the alanine scanning was overestimated, most of the simulation results obtained were in good agreement with those determined by the site-directed mutagenesis. The overestimated free energy difference for R447 may be attributed to the fact that the corresponding solvation energy was overestimated. We suspected that in reality the nearby E450 residue may compensate for the R447A mutant since these two residues are very close to each other (Figure 5).

Our experimental and theoretical results also manifest the facts that residues Y155, H195, and W218 of subsites −2 and −3 may play an important role in interacting with ligand G_3_T. The success of the theoretical study may be ascribed to the facts that (i) the ligand was correctly placed inside the active site and (ii) the structure of protein-ligand complex constructed was appropriately refined through the MD simulation technique. The structure of protein-ligand complex theoretically constructed and refined was also validated through an *ab initio* quantum chemical calculation using the Gaussian09 package. A truncated model system was used in the *ab initio* quantum chemical calculation and the result gave an activation energy that was close to those reported in the literatures for the same type of hydrolysis reactions. These results show that it is feasible to use the combined computational techniques to predict the roles of some important residues involving in a complicated catalytic reaction like the one studied here.

## References

[1] Richards AB, Krakowka S, Dexter LB, Schmid H, Wolterbeek APM, et al (2002) Trehalose: a review of properties, history of use and human tolerance, and results of multiple safety studies. Food and Chemical Toxicology 40: 871–898. 12065209 10.1016/s0278-6915(02)00011-x

[2] Crowe JH (2007) Trehalose as a “chemical chaperone”: Fact and fantasy. Molecular Aspects of the Stress Response: Chaperones, Membranes and Networks 594: 143–158. 10.1007/978-0-387-39975-1_13 17205682

[3] Kato M (1999) Trehalose production with a new enzymatic system from Sulfolobus solfataricus KM1. Journal of Molecular Catalysis B-Enzymatic 6: 223–233.

[4] Fang TY, Tseng WC, Pan CH, Chun YT, Wang MY (2007) Protein engineering of Sulfolobus solfataricus maltooligosyltrehalose synthase to alter its selectivity. Journal of Agricultural and Food Chemistry 55: 5588–5594. 17567140 10.1021/jf0701279

[5] Fang TY, Tseng WC, Shih TY, Wang MY (2008) Identification of the essential catalytic residues and selectivity-related residues of maltooligosyltrehalose trehalohydrolase from the thermophilic archaeon Sulfolobus solfataricus ATCC 35092. Journal of Agricultural and Food Chemistry 56: 5628–5633. 18563901 10.1021/jf073320b

[6] Feese MD, Kato Y, Tamada T, Kato M, Komeda T, et al (2000) Crystal structure of glycosyltrehalose trehalohydrolase from the hyperthermophilic archaeum Sulfolobus solfataricus. Journal of Molecular Biology 301: 451–464. 10926520 10.1006/jmbi.2000.3977

[7] Timmins J, Leiros HKS, Leonard G, Leiros I, McSweeney S (2005) Crystal structure of maltooligosyltrehalose trehalohydrolase from Deinococcus radiodurans in complex with disaccharides. Journal of Molecular Biology 347: 949–963. 15784255 10.1016/j.jmb.2005.02.011

[8] Fang TY, Tseng WC, Guo MS, Shih TY, Hung XG (2006) Expression, purification, and characterization of the maltooligosyltrehalose trehalohydrolase from the thermophilic archaeon Sulfolobus solfataricus ATCC 35092. Journal of Agricultural and Food Chemistry 54: 7105–7112. 16968069 10.1021/jf061318z

[9] Swanson JMJ, Henchman RH, McCammon JA (2004) Revisiting free energy calculations: A theoretical connection to MM/PBSA and direct calculation of the association free energy. Biophysical Journal 86: 67–74. 14695250 10.1016/S0006-3495(04)74084-9 PMC1303837

[10] Kollman PA, Massova I, Reyes C, Kuhn B, Huo SH, et al (2000) Calculating structures and free energies of complex molecules: Combining molecular mechanics and continuum models. Accounts of Chemical Research 33: 889–897. 11123888 10.1021/ar000033j

[11] Weiser J, Shenkin PS, Still WC (1999) Approximate atomic surfaces from linear combinations of pairwise overlaps (LCPO). Journal of Computational Chemistry 20: 217–230.

[12] Sitkoff D, Sharp KA, Honig B (1994) Accurate Calculation of Hydration Free-Energies Using Macroscopic Solvent Models. Journal of Physical Chemistry 98: 1978–1988.

[13] Hawkins GD, Cramer CJ, Truhlar DG (1995) Pairwise Solute Descreening of Solute Charges from a Dielectric Medium. Chemical Physics Letters 246: 122–129.

[14] Hawkins GD, Cramer CJ, Truhlar DG (1996) Parametrized models of aqueous free energies of solvation based on pairwise descreening of solute atomic charges from a dielectric medium. Journal of Physical Chemistry 100: 19824–19839.

[15] Onufriev A, Bashford D, Case DA (2000) Modification of the generalized Born model suitable for macromolecules. Journal of Physical Chemistry B 104: 3712–3720.

[16] Li T, Froeyen M, Herdewijn P (2008) Computational alanine scanning and free energy decomposition for E-coli type I signal peptidase with lipopeptide inhibitor complex. Journal of Molecular Graphics & Modelling 26: 813–823. 17532654 10.1016/j.jmgm.2007.04.007

[17] Kuhn B, Gerber P, Schulz-Gasch T, Stahl M (2005) Validation and use of the MM-PBSA approach for drug discovery. Journal of Medicinal Chemistry 48: 4040–4048. 15943477 10.1021/jm049081q

[18] Luo C, Xu LF, Zheng SX, Luo Z, Jiang XM, et al (2005) Computational analysis of molecular basis of 1: 1 interactions of NRG-1 beta wild-type and variants with ErbB3 and ErbB4. Proteins-Structure Function and Bioinformatics 59: 742–756. 10.1002/prot.20443 15822127

[19] Huo SH, Wang JM, Cieplak P, Kollman PA, Kuntz ID (2002) Molecular dynamics and free energy analyses of cathepsin D-inhibitor interactions: Insight into structure-based ligand design. Journal of Medicinal Chemistry 45: 1412–1419. 11906282 10.1021/jm010338j

[20] Xu Y, Wang RX (2006) A computational analysis of the binding affinities of FKBP12 inhibitors using the MM-PB/SA method. Proteins-Structure Function and Bioinformatics 64: 1058–1068. 10.1002/prot.21044 16838311

[21] Lepsik M, Kriz Z, Havlas Z (2004) Efficiency of a second-generation HIV-1 protease inhibitor studied by molecular dynamics and absolute binding free energy calculations. Proteins-Structure Function and Bioinformatics 57: 279–293. 10.1002/prot.20192 15340915

[22] Kuhn B, Kollman PA (2000) Binding of a diverse set of ligands to avidin and streptavidin: An accurate quantitative prediction of their relative affinities by a combination of molecular mechanics and continuum solvent models. Journal of Medicinal Chemistry 43: 3786–3791. 11020294 10.1021/jm000241h

[23] Wang JM, Morin P, Wang W, Kollman PA (2001) Use of MM-PBSA in reproducing the binding free energies to HIV-1 RT of TIBO derivatives and predicting the binding mode to HIV-1 RT of efavirenz by docking and MM-PBSA. Journal of the American Chemical Society 123: 5221–5230. 11457384 10.1021/ja003834q

[24] Thorsteinsdottir HB, Schwede T, Zoete V, Meuwly M (2006) How inaccuracies in protein structure models affect estimates of protein-ligand interactions: Computational analysis of HIV-1 protease inhibitor binding. Proteins-Structure Function and Bioinformatics 65: 407–423. 10.1002/prot.21096 16941468

[25] Gohlke H, Kiel C, Case DA (2003) Insights into protein-protein binding by binding free energy calculation and free energy decomposition for the Ras-Raf and Ras-RaIGDS complexes. Journal of Molecular Biology 330: 891–913. 12850155 10.1016/s0022-2836(03)00610-7

[26] Zoete V, Meuwly M, Karplus M (2005) Study of the insulin dimerization: Binding free energy calculations and per-residue free energy decomposition. Proteins-Structure Function and Bioinformatics 61: 79–93. 10.1002/prot.20528 16080143

[27] Zoete V, Michielin O (2007) Comparison between computational alanine scanning and per-residue binding free energy decomposition for protein-protein association using MM-GBSA: Application to the TCR-p-MHC complex. Proteins-Structure Function and Bioinformatics 67: 1026–1047. 10.1002/prot.21395 17377991

[28] Massova I, Kollman PA (1999) Computational alanine scanning to probe protein-protein interactions: A novel approach to evaluate binding free energies. Journal of the American Chemical Society 121: 8133–8143.

[29] Huo S, Massova I, Kollman PA (2002) Computational alanine scanning of the 1: 1 human growth hormone-receptor complex. Journal of Computational Chemistry 23: 15–27. 11913381 10.1002/jcc.1153

[30] Villacanas O, Rubio-Martinez J (2006) Reducing CDK4/6-p16(INK4a) interface: Computational alanine scanning of a peptide bound to CDK6 protein. Proteins-Structure Function and Bioinformatics 63: 797–810. 10.1002/prot.20943 16508961

[31] Moreira IS, Fernandes PA, Ramos MJ (2006) Unraveling the importance of protein-protein interaction: Application of a computational alanine-scanning mutagenesis to the study of the IgG1 streptococcal protein G (C2 fragment) complex. Journal of Physical Chemistry B 110: 10962–10969. 10.1021/jp054760d 16771349

[32] Chong LT, Swope WC, Pitera JW, Pande VS (2006) Kinetic computational alanine scanning: Application to p53 oligomerization. Journal of Molecular Biology 357: 1039–1049. 16457841 10.1016/j.jmb.2005.12.083

[33] Zoete V, Meuwly M (2006) Importance of individual side chains for the stability of a protein fold: Computational alanine scanning of the insulin monomer. Journal of Computational Chemistry 27: 1843–1857. 16981237 10.1002/jcc.20512

[34] Tseng WC, Lin JW, Wei TY, Fang TY (2008) A novel megaprimed and ligase-free, PCR-based, site-directed mutagenesis method. Analytical Biochemistry 375: 376–378. 18198125 10.1016/j.ab.2007.12.013

[35] Bradford MM (1976) Rapid and Sensitive Method for Quantitation of Microgram Quantities of Protein Utilizing Principle of Protein-Dye Binding. Analytical Biochemistry 72: 248–254. 942051 10.1016/0003-2697(76)90527-3

[36] Wilkinson AJ, Fersht AR, Blow DM, Winter G (1983) Site-Directed Mutagenesis as a Probe of Enzyme Structure and Catalysis - Tyrosyl-Transfer Rna-Synthetase Cysteine-35 to Glycine-35 Mutation. Biochemistry 22: 3581–3586. 6615786 10.1021/bi00284a007

[37] Pearlman DA, Case DA, Caldwell JW, Ross WS, Cheatham TE, et al (1995) Amber, a Package of Computer-Programs for Applying Molecular Mechanics, Normal-Mode Analysis, Molecular-Dynamics and Free-Energy Calculations to Simulate the Structural and Energetic Properties of Molecules. Computer Physics Communications 91: 1–41.

[38] Case DA, Cheatham TE, Darden T, Gohlke H, Luo R, et al (2005) The Amber biomolecular simulation programs. Journal of Computational Chemistry 26: 1668–1688. 16200636 10.1002/jcc.20290 PMC1989667

[39] Case DA, Cheatham TE, Simmerling CL, Wang J, Duke RE, et al.. (2010) AMBER 11, University of California, San Francisco.

[40] Jones G, Willett P, Glen RC (1995) Molecular Recognition of Receptor-Sites Using a Genetic Algorithm with a Description of Desolvation. Journal of Molecular Biology 245: 43–53. 7823319 10.1016/s0022-2836(95)80037-9

[41] Jones G, Willett P, Glen RC, Leach AR, Taylor R (1997) Development and validation of a genetic algorithm for flexible docking. Journal of Molecular Biology 267: 727–748. 9126849 10.1006/jmbi.1996.0897

[42] Manuel RC, Lloyd RS (1997) Cloning, Overexpression, and Biochemical Characterization of the Catalytic Domain of MutY. Biochemistry 36: 11140–11152. 9287157 10.1021/bi9709708

[43] Anandakrishnan R, Aguilar B, Onufriev AV (2012) H++3.0: automating pK prediction and the preparation of biomolecular structures for atomistic molecular modeling and simulations. Nucleic Acids Research 40: W537–W541. 22570416 10.1093/nar/gks375 PMC3394296

[44] Olsson MHM, Sondergaard CR, Rostkowski M, Jensen JH (2011) PROPKA3: Consistent Treatment of Internal and Surface Residues in Empirical pK (a) Predictions. Journal of Chemical Theory and Computation 7: 525–537. 26596171 10.1021/ct100578z

[45] Hornak V, Abel R, Okur A, Strockbine B, Roitberg A, et al (2006) Comparison of multiple Amber force fields and development of improved protein backbone parameters. Proteins-Structure Function and Bioinformatics 65: 712–725. 10.1002/prot.21123 PMC4805110 16981200

[46] Kirschner KN, Yongye AB, Tschampel SM, Gonzalez-Outeirino J, Daniels CR, et al (2008) GLYCAM06: a generalizable biomolecular force field. Carbohydrates. Journal of Computational Chemistry 29: 622–655. 17849372 10.1002/jcc.20820 PMC4423547

[47] Jorgensen WL (1982) Quantum and Statistical Mechanical Studies of Liquids .24. Revised Tips for Simulations of Liquid Water and Aqueous-Solutions. Journal of Chemical Physics 77: 4156–4163.

[48] Jorgensen WL, Chandrasekhar J, Madura JD, Impey RW, Klein ML (1983) Comparison of Simple Potential Functions for Simulating Liquid Water. Journal of Chemical Physics 79: 926–935.

[49] Darden T, York D, Pedersen L (1993) Particle Mesh Ewald – an N.Log(N) Method for Ewald Sums in Large Systems. Journal of Chemical Physics 98: 10089–10092.

[50] Miyamoto S, Kollman PA (1992) Settle – an Analytical Version of the Shake and Rattle Algorithm for Rigid Water Models. Journal of Computational Chemistry 13: 952–962.

[51] GelAnalyzer 2010. Available: http://www.gelanalyzer.com/download.html. Accessed 2013 May 16.

[52] Fersht AR, Shi JP, Knilljones J, Lowe DM, Wilkinson AJ, et al (1985) Hydrogen-Bonding and Biological Specificity Analyzed by Protein Engineering. Nature 314: 235–238. 3845322 10.1038/314235a0

[53] Saharay M, Guo H, Smith JC (2010) Catalytic mechanism of cellulose degradation by a cellobiohydrolase, CelS. PLoS One 5: e12947. 20967294 10.1371/journal.pone.0012947 PMC2953488

[54] Uma Maheswar Rao JL, Satyanarayana T (2007) Purification and characterization of a hyperthermostable and high maltogenic alpha-amylase of an extreme thermophile Geobacillus thermoleovorans. Appl Biochem Biotechnol 142: 179–193. 18025579 10.1007/s12010-007-0017-4

[55] Mishra RS, Maheshwari R (1996) Amylases of the thermophilic fungus Thermomyces lanuginosus: Their purification, properties, action on starch and response to heat. Journal of Biosciences 21: 653–672.

